# Association between dietary carotenoids intake and fecal incontinence in American adults: evidence from NAHNES 2005–2010

**DOI:** 10.3389/fnut.2024.1486741

**Published:** 2024-11-20

**Authors:** Zhigang Li, Zan Wen, Jiaqing Cao, Fei Cheng

**Affiliations:** The Second Affiliated Hospital, Jiangxi Medical College, Nanchang University, Nanchang, Jiangxi, China

**Keywords:** dietary carotenoids intake, fecal incontinence, NHANES, *β*-carotene, lutein/zeaxanthin

## Abstract

**Object:**

Carotenoids represent a class of bioactive compounds with potential implications for gut health. However, the relationship between dietary carotenoid intake (DCI) and fecal incontinence (FI) remains unclear. This study aims to elucidate the association between DCI and the risk of FI.

**Methods:**

Participants aged 20 and above from the National Health and Nutrition Examination Survey (NHANES, 2005–2010) were included in the study. Data on FI were derived from the bowel health questionnaire, while DCI information was obtained from dietary interviews. Survey-weighted logistic regression analysis and restricted cubic splines (RCS) were employed to evaluate the relationship between DCI, its subtypes, and FI. Weighted quantile sum (WQS) regression was utilized to assess the overall effect of DCI and its predominant subtypes. Finally, subgroup analyses were conducted.

**Result:**

The study included a total of 11,915 participants, of whom 1,023 (7.0%) experienced FI. Logistic regression analysis revealed that, after adjusting for all covariates, there was a significant inverse association between DCI and the risk of FI (Model 2: Q4 vs. Q1, OR = 0.67, 95% CI: 0.52–0.86, *p* = 0.003). However, among the DCI subtypes, only *β*-carotene was found to have a significant inverse relationship with FI (Model 2: Q4 vs. Q1, OR = 0.68, 95% CI: 0.52–0.88, *p* = 0.005). The RCS curves indicated no non-linear relationship between DCI, its subtypes, and FI (all *p*-non-linear >0.05). WQS analysis identified *β*-carotene (weight 38.2%) and lutein/zeaxanthin (weight 27.8%) as the primary contributors.

**Conclusion:**

High levels of carotenoid intake, particularly *β*-carotene and lutein/zeaxanthin, are associated with a reduced risk of fecal incontinence. This discovery provides dietary recommendations for patients suffering from FI.

## Introduction

Fecal incontinence (FI) is a prevalent gastrointestinal disorder characterized by the involuntary leakage of solid or liquid stool (1). The global prevalence of FI is reported to be approximately 8%, with a higher incidence observed among the elderly and women (2). However, the actual prevalence of FI is likely underestimated due to factors such as underreporting by patients and the avoidance of medical consultation (3, 4). FI not only severely impacts quality of life but is also associated with a range of comorbidities, including gastrointestinal cancers, lymphoma, depression, and sarcopenia (5–8). Therefore, identifying risk factors for FI and implementing preventive measures is crucial.

An increasing number of studies have indicated that dietary patterns are closely related to the occurrence of FI. An early prospective study reported that individuals in the FI group consumed higher amounts of carbohydrates, manganese, and vitamin B1 compared to the control group (9). Additionally, certain dietary fibers, such as psyllium, have been shown to reduce the risk of FI (10, 11). Conversely, limiting the intake of foods rich in fermentable oligosaccharides, disaccharides, monosaccharides, and polyols (FODMAPs) may help alleviate FI symptoms (12, 13). This connection may be attributed to dietary patterns influencing gut microbiota, triggering gastrointestinal inflammatory responses, and causing sensory and neural impairments (14).

Carotenoids are a class of natural lipophilic pigments synthesized by plants, algae, and cyanobacteria. In daily life, individuals primarily obtain these pigments through the consumption of vegetables and fruits such as tomatoes, carrots, and spinach (15). Previous research has indicated that carotenoids possess antioxidant and anti-inflammatory properties, which can reduce the risk of obesity, cardiovascular diseases, and cancer, thereby promoting human health (16–18). Moreover, carotenoids also play a positive role in gastrointestinal diseases. A recent cross-sectional study found that dietary lycopene intake is associated with the prevention of constipation in men, while *α*-carotene intake is linked to a reduced risk of constipation in women (19). Diets rich in carotenoids may help prevent small intestinal colitis and gastric ulcers, as well as alleviate symptoms in patients with ulcerative colitis (20, 21). Additionally, research by Leenders et al. (22) has identified a correlation between *β*-carotene and colorectal cancer. Furthermore, previous studies have also reported that the metabolite of carotenoids, all-trans retinoic acid, plays roles in reducing intestinal inflammation, regulating intestinal microbiota and modulating intestinal immune responses (23).

However, as a common gastrointestinal disorder, few studies have examined the relationship between dietary carotenoid intake (DCI) and FI. Therefore, the current study aims to explore the association between DCI and the risk of FI using a large-scale population cohort database (NHANES). We hypothesize that higher DCI is associated with a reduced risk of FI.

## Methods

### Data availability and study population

The NHANES database, conducted by the National Center for Health Statistics (NCHS) under the auspices of the Centers for Disease Control and Prevention (CDC), is a large-scale program designed to assess the health and nutritional status of adults and children in the United States. This survey is conducted biennially, employing complex probability sampling methods to select representative samples from counties across the nation for comprehensive health and nutritional evaluations. The data for this study were derived from the NHANES survey cycles of 2005–2006, 2007–2008, and 2009–2010. which are publicly available and can be accessed through the CDC’s official website (NHANES – National Health and Nutrition Examination Survey Homepage^1^). We selected these three cycles because, during this period, the Bowel Health Questionnaire (BHQ) meticulously recorded information pertinent to fecal incontinence (FI).

**Figure 1:**
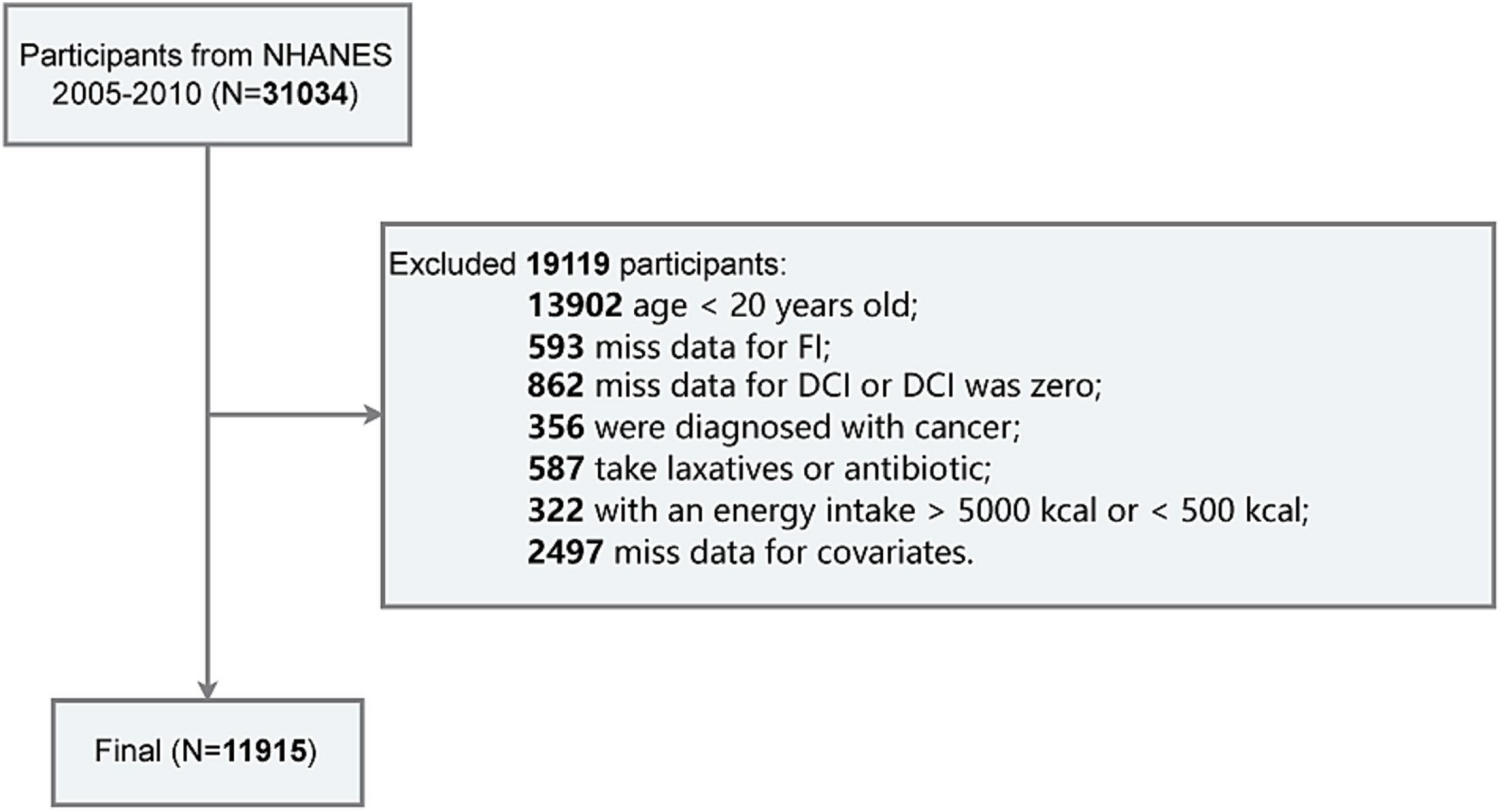
Flowchart of the study design and participants.

The exclusion criteria for this study are as follows (Figure 1): (1) age under 20 years (*n* = 13,902); (2) missing data for FI (*n* = 593); (3) missing data for DCI or DCI equal to zero (*n* = 862); (4) diagnosed with cancer (*n* = 356); (5) recent use of laxatives or antibiotics (*n* = 587); (6) total energy intake exceeding 5,000 kcal or less than 500 kcal (*n* = 322); and (7) missing data for covariates such as PIR, education level, BMI, smoking status, alcohol consumption, hypertension, or diabetes (*n* = 2,497). Finally, a total of 11,915 participants were included in the final analysis.

### Definition of FI

In the NHANES database, the Bowel Health Questionnaire (BHQ) provides detailed personal interview data on FI and bowel function for adults aged 20 years and older. Conducted in mobile examination centers (MECs), participants were queried about the components of involuntary stool discharge (solid, liquid, mucus, or gas) and the frequency of these episodes over the past 30 days. Additionally, participants utilized the Bristol Stool Form Scale (BSFS) cards to assess stool consistency. In this study, FI was defined as at least one episode of involuntary discharge of solid, mucus-like, or liquid stool within a month, excluding gas leakage.

### Measurement of DCI

To obtain dietary data from participants, the United States Department of Agriculture (USDA) and the United States Department of Health and Human Services (DHHS) conducted two dietary interviews. The first interview was conducted in the MEC, and the second interview was carried out via telephone 3–10 days after the first interview. All procedures were administered by well-trained personnel. The nutritional and energy calculations for each consumed food or beverage were derived using the USDA’s Food and Nutrition Database for Dietary Studies (FNDDS), and food group classifications adhered to the guidelines of the Food Patterns Equivalent Database (FPED) provided by USDA.

To ensure data accuracy, this study utilized the dietary data from the first day of interviews. Consequently, we extracted the total DCI and its subtypes, including *α*-carotene, *β*-carotene, *β*-cryptoxanthin, lycopene, and lutein/zeaxanthin, from the “DR1TOT” file in the NHANES dietary data module. These data were natural log-transformed (ln-transformed) and categorized into quartiles for analysis, as detailed in Supplementary Table S1.

### Covariates

Based on previous research, we included several important variables that could potentially influence FI as covariates. These variables encompass demographic characteristics [age, sex, race, education level, and poverty income ratio (PIR)], BMI, daily behaviors (smoking status and alcohol use), chronic diseases (hypertension and diabetes), and total energy intake (24–27).

Race was categorized as Non-Hispanic White, Non-Hispanic Black, Mexican American, and other races (including other Hispanics and multiple races). Education level was divided into less than high school, high school, and college or above. BMI classifications were normal (<25 kg/m^2^), overweight (25–30 kg/m^2^), and obese (>30 kg/m^2^). PIR was dichotomized at the median value (2.25). Smoking status was defined as: never smoking (fewer than 100 cigarettes in their lifetime), former smoking (more than 100 cigarettes in their lifetime, not currently smoking), and current smoking (more than 100 cigarettes in their lifetime, currently smoking). Alcohol use was categorized as: never drink (fewer than 12 drinks in their lifetime), former drink (≥12 drinks in their lifetime, not in the past year), and current drink (more than 12 drinks in their lifetime, currently drinking). Participants were considered to have hypertension if they were told by a doctor that you have high blood pressure, or use antihypertensive medication, or their blood pressure > 140/90 mmHg. Diabetes was defined by a doctor’s diagnosis, HbA1c ≥6.5%, fasting glucose ≥7.0 mmol/L, random glucose ≥11.1 mmol/L, 2-h OGTT glucose ≥11.1 mmol/L, or use of antidiabetic medication or insulin.

### Statistical analysis

According to the NHANES analysis guidelines, sample weights, strata, and primary sampling units (PSU) were used to account for the complex survey design. Continuous variables were presented as weighted means (± standard error), and categorical variables were shown as unweighted counts (weighted percentages). Comparisons between continuous variables employed *t*-tests, while comparisons between categorical variables used chi-square tests. Weighted logistic regression analysis was utilized to evaluate the relationship between DCI and FI. To evaluate the relationship between DCI and FI, three graded incremental logistic models were constructed: Model 1 was unadjusted; Model 2 adjusted for age, sex, race, education level, and PIR; and Model 3 further adjusted for BMI, smoking status, alcohol use, hypertension, diabetes, and total dietary energy intake. Additionally, restricted cubic splines (RCS) were employed to assess the dose–response relationship between DCI and FI.

The Weighted Quantile Sum (WQS) regression model is a statistical method used to evaluate the association between mixed exposures and health outcomes (28). In this study, WQS was employed to assess the overall effect of DCI and to identify key DCI subtypes contributing to the occurrence of FI by calculating their weights. Specifically, we established a WQS index related to FI outcomes based on the quartiles of DCI and divided participants into training and validation sets in a 4:6 ratio, employing 1,000 bootstrap samples to ensure result stability. The WQS model adjusted for all variables in Model 2, with DCI subtypes having weights ≥0.2 considered significant contributors. Lastly, we conducted subgroup analyses and interaction tests for each variable in Model 2 to explore the negative correlation between DCI and FI across different subgroups.

All analyses in this study were conducted using R version 4.3.2 (R: The R Project for Statistical Computing^2^), utilizing the “survey,” “rms,” and “gWQS” packages for weighted analysis, RCS analysis, and WQS analysis, respectively. A two-sided *p*-value of less than 0.05 was considered statistically significant.

## Results

### Baseline characteristics

A total of 11,915 participants were included in this study, representing an estimated total population of 171,540,058 individuals. The average age was 46.7 (±0.4) years, with males comprising 49.4% (6,112 individuals). Among them, 1,023 participants (8.6%) experienced fecal incontinence (FI). FI was more prevalent among older individuals, females, Non-Hispanic White individuals, those with higher BMI, drinkers, individuals with hypertension, and those with diabetes. Furthermore, FI patients had lower DCI compared to normal group (8.48 vs. 8.64, *p* = 0.025). However, among the five DCI subclasses, only lycopene showed a statistically significant difference between the two groups (5.61 vs. 6.04, *p* = 0.010). Although the intake of *α*-carotene, *β*-carotene, *β*-cryptoxanthin, and lutein/zeaxanthin was lower in FI patients compared to those without FI, these differences were not statistically significant (Table 1).

**Table 1:** Baseline characteristics of normal and participants with FI.

Characteristics^a^	Overall, *N* = 11,915	Normal, *N =* 10,892	FI, *N* = 1,023	*p-*value
Age, year	46.7 (±0.4)	46.0 (±0.3)	55.2 (±0.6)	**<0.001**
Gender				**0.041**
Female	5,803 (50.6%)	5,255 (50.1%)	548 (55.5%)	
Male	6,112 (49.4%)	5,637 (49.9%)	475 (44.5%)	
Race				**0.002**
Non-Hispanic White	6,005 (72.4%)	5,399 (71.9%)	606 (78.0%)	
Non-Hispanic Black	2,376 (10.7%)	2,189 (10.8%)	187 (9.4%)	
Mexican American	2,109 (7.8%)	1,983 (8.0%)	126 (4.8%)	
Other races	1,425 (9.2%)	1,321 (9.3%)	104 (7.8%)	
Education level				0.355
Less than high school	3,240 (17.2%)	2,937 (17.1%)	303 (19.1%)	
High school	2,855 (24.5%)	2,610 (24.5%)	245 (24.6%)	
College or above	5,820 (58.3%)	5,345 (58.5%)	475 (56.3%)	
PIR				0.058
≤2.25	5,964 (36.9%)	5,410 (36.6%)	554 (40.7%)	
>2.25	5,951 (63.1%)	5,482 (63.4%)	469 (59.3%)	
BMI, kg/m^2^				**0.003**
Continuous	28.7 (±0.1)	28.6 (±0.1)	30.0 (±0.4)	**<0.001**
<25	3,452 (31.7%)	3,193 (32.1%)	259 (27.5%)	
25–30	4,085 (33.3%)	3,764 (33.6%)	321 (29.8%)	
>30	4,378 (35.0%)	3,935 (34.3%)	443 (42.7%)	
Smoking status				0.061
Never	6,241 (52.7%)	5,777 (53.1%)	464 (47.2%)	
Former	3,044 (24.7%)	2,717 (24.3%)	327 (28.8%)	
Current	2,630 (22.7%)	2,398 (22.6%)	232 (24.0%)	
Alcohol use				**<0.001**
Never	1,538 (10.4%)	1,399 (10.2%)	139 (12.4%)	
Former	2,354 (16.3%)	2,094 (15.8%)	260 (22.1%)	
Current	8,023 (73.3%)	7,399 (74.0%)	624 (65.5%)	
Hypertension				**<0.001**
No	6,981 (63.5%)	6,533 (64.8%)	448 (47.8%)	
Yes	4,934 (36.5%)	4,359 (35.2%)	575 (52.2%)	
Diabetes				**<0.001**
No	9,847 (87.6%)	9,091 (88.2%)	756 (80.5%)	
Yes	2,068 (12.4%)	1,801 (11.8%)	267 (19.5%)	
Energy intake, kcal	2,134.27 (±14.33)	2,136.38 (±15.31)	2,110.02 (±43.73)	0.461
ln(*α*-carotene, mcg)	3.89 (±0.04)	3.90 (±0.04)	3.85 (±0.12)	0.717
ln(*β*-carotene, mcg)	6.67 (±0.03)	6.67 (±0.03)	6.61 (±0.07)	0.308
ln(*β*-cryptoxanthin, mcg)	3.10 (±0.04)	3.11 (±0.04)	3.08 (±0.10)	0.811
ln(lycopene, mcg)	6.01 (±0.06)	6.04 (±0.06)	5.61 (±0.17)	**0.010**
ln(lutein/zeaxanthin, mcg)	6.49 (±0.03)	6.50 (±0.03)	6.41 (±0.07)	0.277
ln(carotenoids, mcg)	8.62 (±0.03)	8.64 (±0.03)	8.48 (±0.07)	**0.025**

FI, fecal Incontinence; PIR, ratio of family income to poverty; BMI, body mass index. ^a^Mean (±se) for continuous and *n*(%) for categorical variables. Bold: *p* < 0.05.

### Multivariable logistic regression

Logistic regression analysis revealed a negative association between DCI and FI (Crude model: Q4 vs. Q1, OR = 0.74, 95% CI: 0.59–0.93, *p* = 0.011, *p* for trend = 0.033). After adjusting for potential confounders, this relationship remained consistent (Model 1: Q4 vs. Q1, OR = 0.71, 95% CI: 0.56–0.90, *p* = 0.006, *p* for trend = 0.014; Model 2: OR = 0.67, 95% CI: 0.52–0.86, *p* = 0.003, *p* for trend = 0.006) (Table 2). However, in univariate analysis (Crude model), no significant associations were found between any DCI subtypes and FI. Only in Model 1 and Model 2 did *β*-carotene show a significant association with FI (Model 1: OR = 0.70, 95% CI: 0.54–0.90, *p* = 0.008, *p* for trend = 0.006; Model 2: OR = 0.68, 95% CI: 0.52–0.88, *p* = 0.005, *p* for trend = 0.004), with a trend analysis indicating a correlation between Lutein/Zeaxanthin and FI (*p* for trend = 0.036).

**Table 2:** Association between DCI and FI.

Ln(DCI, mcg)	Crude model^a^		Model 1^a^		Model 2^a^	
	OR(95% CI)	*p-*value	OR(95% CI)	*p-*value	OR(95% CI)	*p-*value
Carotenoids						
Q1	Ref		Ref		ref	
Q2	0.84(0.65,1.08)	0.159	0.85(0.65,1.11)	0.234	0.83(0.63,1.08)	0.158
Q3	0.89(0.65,1.21)	0.431	0.89(0.65,1.23)	0.480	0.86(0.63,1.17)	0.331
Q4	0.74(0.59,0.93)	**0.011**	0.71(0.56,0.90)	**0.006**	0.67(0.52,0.86)	**0.003**
*p* for trend		**0.033**		**0.014**		**0.006**
*α*-carotene						
Q1	Ref		Ref		Ref	
Q2	0.93(0.69,1.25)	0.621	0.86(0.62,1.18)	0.344	0.86(0.62,1.21)	0.370
Q3	0.85(0.64,1.13)	0.264	0.76(0.56,1.02)	0.063	0.75(0.55,1.01)	0.054
Q4	1.02(0.76,1.36)	0.903	0.83(0.61,1.12)	0.219	0.84(0.61,1.15)	0.259
*p* for trend		0.946		0.159		0.181
*β*-carotene						
Q1	Ref		Ref		Ref	
Q2	0.89(0.67,1.19)	0.438	0.87(0.65,1.18)	0.361	0.83(0.61,1.12)	0.206
Q3	0.92(0.69,1.21)	0.525	0.84(0.64,1.11)	0.212	0.79(0.60,1.03)	0.082
Q4	0.84(0.65,1.07)	0.158	0.70(0.54,0.90)	**0.008**	0.68(0.52,0.88)	**0.005**
*p* for trend		0.194		**0.006**		**0.004**
*β*-cryptoxanthin						
Q1	Ref		Ref		Ref	
Q2	0.95(0.73,1.25)	0.718	0.92(0.70,1.21)	0.534	0.90(0.68,1.18)	0.424
Q3	1.07(0.79,1.44)	0.652	0.98(0.72,1.34)	0.915	0.97(0.70,1.33)	0.834
Q4	0.93(0.69,1.25)	0.617	0.79(0.58,1.06)	0.116	0.78(0.58,1.06)	0.108
*p* for trend		0.835		0.197		0.189
Lycopene						
Q1	Ref		Ref		Ref	
Q2	0.86(0.65,1.14)	0.297	0.89(0.67,1.19)	0.429	0.89(0.66,1.18)	0.393
Q3	0.78(0.61,1.00)	0.052	0.87(0.67,1.12)	0.274	0.86(0.67,1.11)	0.238
Q4	0.79(0.61,1.02)	0.065	0.90(0.70,1.17)	0.435	0.85(0.65,1.12)	0.230
*p* for trend		0.052		0.431		0.237
Lutein/zeaxanthin						
Q1	Ref		Ref		Ref	
Q2	1.09(0.81,1.45)	0.563	1.01(0.75,1.37)	0.945	0.97(0.71,1.33)	0.845
Q3	1.02(0.77,1.36)	0.862	0.94(0.70,1.26)	0.658	0.89(0.66,1.21)	0.448
Q4	0.86(0.63,1.17)	0.341	0.76(0.56,1.05)	0.090	0.73(0.52,1.02)	0.061
*p* for trend		0.274		0.059		**0.036**

OR = odds ratio; CI = confidence interval. ^a^Crude model, unadjusted model; model 1, age, gender, race, education level, and PIR were adjusted; model 2, age, gender, race, education level, PIR, BMI, energy intake, smoking status, alcohol use, hypertension, and diabetes were adjusted. Bold: *p* < 0.05.

### RCS

**Figure 2:**
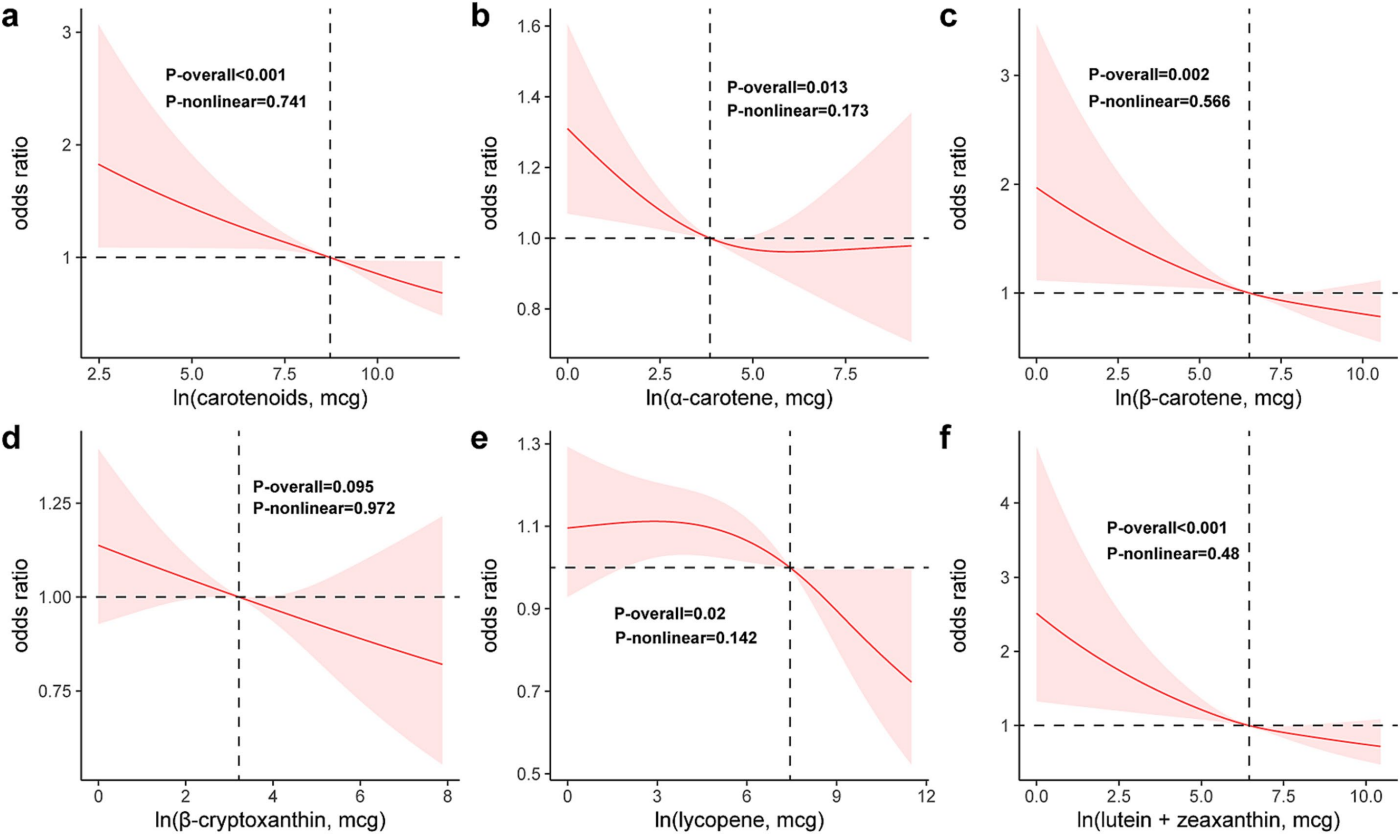
Restricted cubic spline model of the odds ratios of FI with DCI (a) and its subclasses: *α*-carotene (b), *β*-carotene (c), *β*-cryptoxanthin (d), lycopene (e), lutein/zeaxanthin (f). All were adjusted for age, gender, race, education level, PIR, BMI, energy intake, smoking status, alcohol use, hypertension, and diabetes.

As illustrated in Figure 2, RCS curves demonstrate a continuous downward trend in the risk of fecal incontinence (FI) with increasing DCI, indicating no non-linear relationship between the two variables (*p*-non-linear = 0.741) (Figure 2a). Similarly, none of the five DCI subtypes showed a non-linear association with FI, and the risk of FI decreased with higher intake levels (Figures 2b–f).

### WQS

**Figure 3:**
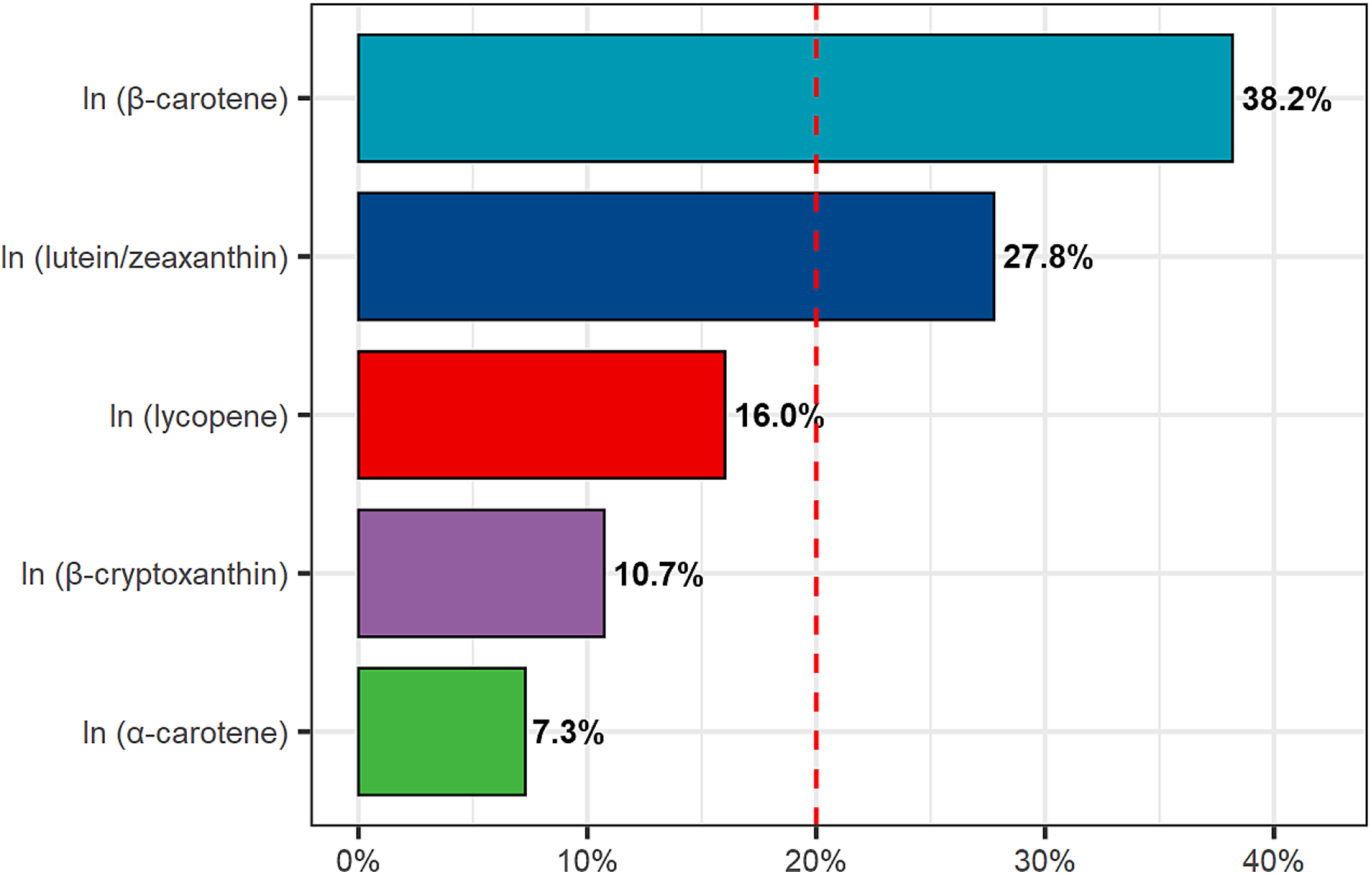
Weighted quantile sum (WQS) model regression index weights. Age, gender, race, education level, PIR, BMI, energy intake, smoking status, alcohol use, hypertension, and diabetes were adjusted.

The WQS regression analysis reveals a negative correlation between DCI and FI (estimated = −8.075e-03, *p* = 0.048). Furthermore, we conducted a detailed analysis of the individual contributions of dietary carotenoids to the combined protective effect. Among these, *β*-carotene (38.2%) exhibited the highest weight, followed by lutein/zeaxanthin (27.8%), lycopene (16.0%), and *β*-cryptoxanthin (10.7%), whereas *α*-carotene (7.3%) showed the lowest weight (Figure 3).

### Stratified analysis

**Figure 4:**
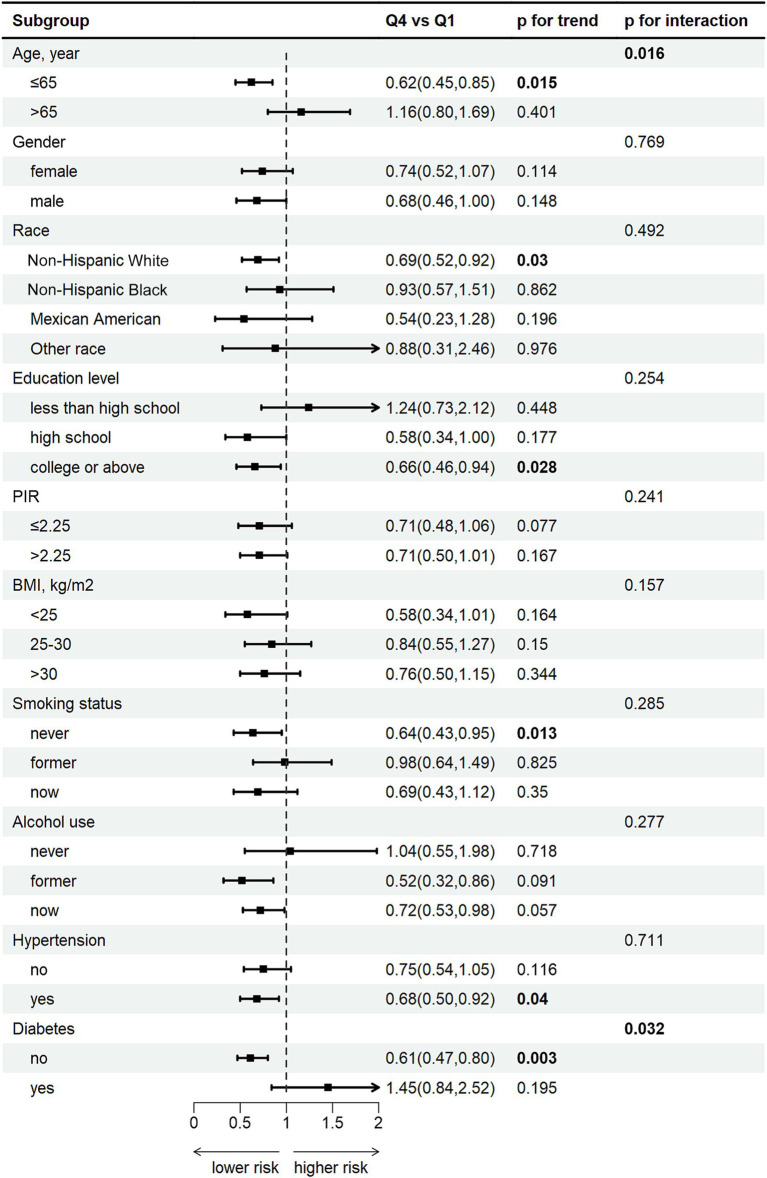
Subgroup analysis according to Q4 of DCI.

Subgroup analysis show a pronounced negative correlation between DCI and FI among individuals 0aged younger than 65, females, Non-Hispanic White individuals, those with a college education or above, non-smokers, individuals with hypertension, and those without diabetes (all *p* < 0.05) (Figure 4). Additionally, age (*p* for interaction = 0.016) and diabetes (*p* for interaction = 0.032) were significant interacting factors affecting the relationship between DCI and FI. Complete subgroup analysis results are detailed in Supplementary Table S2.

## Discussion

Previous studies have elucidated the significant role of dietary patterns in the treatment and prevention of FI. A prospective study from the Nurses’ Health Study revealed a correlation between pro-inflammatory diets and an increased risk of FI in elderly women (29). A cross-sectional survey from the NHANES, which calculated the Dietary Inflammatory Index (DII) for 27 food items, also found that a higher DII was associated with an increased risk of FI (30). Moreover, research by Andy U demonstrated that structured dietary interventions could ameliorate FI symptoms in elderly women (31). However, despite the efficacy of dietary interventions as a treatment modality for FI, there remains a lack of definitive evidence regarding which specific dietary components are most beneficial for ameliorating FI.

Based on a large, nationally representative cohort, we identified an inverse association between DCI and the risk of FI. To evaluate the relationship between DCI and FI, we constructed three graded incremental logistic models. After adjusting for all confounding variables, DCI maintained a robust inverse association with FI (Model 2: OR = 0.67, *p* = 0.003). The WQS analysis also indicated a negative correlation between DCI and FI (estimate = −8.075e-03, *p* = 0.048), with *β*-carotene (38.2%) and lutein/zeaxanthin (27.8%) contributing most significantly. Furthermore, RCS curves suggested a linear inverse relationship between DCI and its subclasses with FI. Subgroup analysis revealed that this inverse relationship was more pronounced among individuals younger than 65 years, females, Non-Hispanic White individuals, those with a college or higher education, non-smokers, hypertensives, and non-diabetics. To our knowledge, this is the first study utilizing a large population cohort to investigate the relationship between dietary carotenoids and FI.

Previous research has indicated that intestinal disorders, particularly diarrhea, rectal emergency symptoms, and chronic disease burden are the most potent independent risk factors for FI in the community. Neurological diseases and inflammatory bowel diseases are also associated with FI. The pathophysiological mechanisms responsible for FI include diarrhea, weakness of the anus and pelvic floor, decreased rectal compliance, and either reduced or increased rectal sensation (1). Several potential mechanisms may elucidate the protective effects of DCI. Firstly, carotenoids exhibit anti-inflammatory and antioxidant properties that can alleviate intestinal inflammation (32). As prominent dietary antioxidants, carotenoids protect the host from oxidative stress and damage inflicted by reactive oxygen species (ROS) (33). Numerous studies have shown that carotenoids such as *β*-carotene, lycopene, and lutein can diminish ROS production, thus exerting anti-inflammatory effects (34–36). Secondly, carotenoids may modulate the gut microbiota, thereby lowering the risk of chronic diseases. A significant portion of ingested carotenoids reaches the colon (37), where they interact with the gut microbiota. This interaction includes upregulating the activity of bifidobacteria and enhancing the abundance of lactobacilli (38, 39). Thirdly, carotenoids can improve intestinal barrier function by increasing the expression of tight junction proteins like occluding and claudin-1, boosting mucus production, and potentially altering gene expression. These enhancements contribute to their antioxidative and anti-inflammatory actions (40, 41). Fourthly, the consumption of carotenoids results in an elevation of all-trans retinoic acid, a metabolite of vitamin A. All-trans retinoic acid plays a pivotal role in the generation of gut-homing lymphocytes and immunoglobulin A antibody-secreting cells (IgA-ASCs), as well as in the differentiation of regulatory T cells. Consequently, these actions are conducive to maintaining intestinal mucosal immune homeostasis and tolerance (42, 43). The anti-inflammatory property of carotenoids can reduce intestinal inflammatory responses, helping to maintain the integrity of the intestinal mucosa and protect nerve cells from inflammatory damage. The antioxidant property can neutralize free radicals to reduce the damage of oxidative stress to intestinal tissues and muscles. In addition, carotenoids regulating the intestinal microbiota can help reduce the risk of chronic diseases and alleviate intestinal dysfunction. Moreover, it maintains intestinal stability by improving intestinal barrier function and regulating immune homeostasis and tolerance.

This study has several strengths. Firstly, it benefits from a substantial sample size derived from a nationally representative population survey. Secondly, the scientific sampling design of NHANES, coupled with comprehensive health and nutrition data, allowed us to adjust for numerous potential confounders, thereby enhancing the reliability of our results. However, the study also has limitations. First, as a cross-sectional study, we cannot establish a causal relationship between DCI and FI. Second, the specific bioavailability of carotenoids remains unknown. Third, since both DCI and FI data were obtained through interviews, there is a potential for recall bias. Fourth, due to data limitations, we cannot exclude other potential factors related to FI.

## Conclusion

Consuming a high dietary intake of carotenoids, particularly *β*-carotene and lutein/zeaxanthin, is negatively associated with the risk of FI, especially among individuals under 65 years of age, females, Non-Hispanic White individuals, those with a college education or higher, non-smokers, individuals with hypertension, and those without diabetes. A high DCI diet may be recommended as a dietary strategy to prevent FI, although further research is needed to elucidate the specific mechanisms.

## Data Availability

Publicly available datasets were analyzed in this study. This data can be found at: NHANES – National Health and Nutrition Examination Survey Homepage: https://www.cdc.gov/nchs/nhanes/index.htm.

## Footnotes

^1^ https://www.cdc.gov/

^2^ https://www.r-project.org
