# Supplementary material for: Association between dietary carotenoids intake and fecal incontinence in American adults: evidence from NAHNES 2005–2010
Source: Front Nutr. 2024 Nov 20;11:1486741. doi: 10.3389/fnut.2024.1486741 (PMC11616493; doi:10.3389/fnut.2024.1486741)
Supplement: Supplementary file 1 [file Table_1.DOCX]

**Table S1.** Quantiles of DCI and its subtypes.

| **Ln (DCI, mcg)** | **Quantile** | **Interval** | **Count** |
| --- | --- | --- | --- |
| **Carotenoids** | Quantile 1 | [1.61, 7.7] | 3001 |
|  | Quantile 2 | (7.7, 8.72] | 2964 |
|  | Quantile 3 | (8.72, 9.56] | 2979 |
|  | Quantile 4 | (9.56, 12.6] | 2971 |
|  |  |  |  |
| **α-carotene** | Quantile 1 | [0, 2.48] | 3074 |
|  | Quantile 2 | (2.48, 3.85] | 2895 |
|  | Quantile 3 | (3.85, 5.49] | 2974 |
|  | Quantile 4 | (5.49, 10.12] | 2972 |
|  |  |  |  |
| **β-carotene** | Quantile 1 | [0, 5.63] | 2990 |
|  | Quantile 2 | (5.63, 6.53] | 2988 |
|  | Quantile 3 | (6.53, 7.67] | 2972 |
|  | Quantile 4 | (7.67, 11.03] | 2965 |
|  |  |  |  |
| **β-cryptoxanthin** | Quantile 1 | [0, 1.79] | 3133 |
|  | Quantile 2 | (1.79, 3.22] | 2878 |
|  | Quantile 3 | (3.22, 4.61] | 2928 |
|  | Quantile 4 | (4.61, 8.9] | 2976 |
|  |  |  |  |
| **Lycopene** | Quantile 1 | [0, 0] | 3008 |
|  | Quantile 2 | (0, 7.44] | 2952 |
|  | Quantile 3 | (7.44, 8.71] | 2987 |
|  | Quantile 4 | (8.71, 12.45] | 2968 |
|  |  |  |  |
| **Lutein/zeaxanthin** | Quantile 1 | [0, 5.75] | 3009 |
|  | Quantile 2 | (5.75, 6.45] | 2986 |
|  | Quantile 3 | (6.45, 7.12] | 2956 |
|  | Quantile 4 | (7.12, 11.57] | 2964 |
