# Supplementary material for: Association between dietary carotenoids intake and fecal incontinence in American adults: evidence from NAHNES 2005–2010
Source: Front Nutr. 2024 Nov 20;11:1486741. doi: 10.3389/fnut.2024.1486741 (PMC11616493; doi:10.3389/fnut.2024.1486741)
Supplement: Supplementary file 2 [file Table_2.docx]

**Table S2.** Subgroup analysis.

| **Subgroup** | **Q1** | **Q2** | **Q3** | **Q4** | ***p* for trend** | ***p* for interaction** |
| --- | --- | --- | --- | --- | --- | --- |
| **Age, year** |  |  |  |  |  | **0.016** |
| ≤65 | ref | 0.74(0.54,1.00) | 0.80(0.56,1.15) | 0.62(0.45,0.85) | **0.015** |  |
| >65 | ref | 1.23(0.80,1.89) | 1.31(0.83,2.07) | 1.16(0.80,1.69) | 0.401 |  |
| **Gender** |  |  |  |  |  | 0.769 |
| female | ref | 0.92(0.65,1.30) | 0.93(0.65,1.33) | 0.74(0.52,1.07) | 0.114 |  |
| male | ref | 0.70(0.48,1.03) | 0.83(0.50,1.38) | 0.68(0.46,1.00) | 0.148 |  |
| **Race** |  |  |  |  |  | 0.492 |
| non-Hispanic white | ref | 0.75(0.54,1.05) | 0.82(0.59,1.14) | 0.69(0.52,0.92) | **0.03** |  |
| non-Hispanic black | ref | 1.43(0.94,2.19) | 1.32(0.77,2.27) | 0.93(0.57,1.51) | 0.862 |  |
| Mexican American | ref | 0.55(0.25,1.21) | 0.55(0.24,1.25) | 0.54(0.23,1.28) | 0.196 |  |
| Other races | ref | 1.27(0.55,2.93) | 1.57(0.55,4.49) | 0.88(0.31,2.46) | 0.976 |  |
| **Education level** |  |  |  |  |  | 0.254 |
| less than high school | ref | 1.42(0.92,2.20) | 1.32(0.81,2.14) | 1.24(0.73,2.12) | 0.448 |  |
| high school | ref | 0.71(0.40,1.25) | 1.01(0.59,1.71) | 0.58(0.34,1.00) | 0.177 |  |
| college or above | ref | 0.73(0.50,1.07) | 0.74(0.52,1.06) | 0.66(0.46,0.94) | **0.028** |  |
| **PIR** |  |  |  |  |  | 0.241 |
| ≤2.25 | ref | 1.05(0.74,1.48) | 0.92(0.62,1.38) | 0.71(0.48,1.06) | 0.077 |  |
| >2.25 | ref | 0.68(0.47,0.98) | 0.85(0.55,1.31) | 0.71(0.50,1.01) | 0.167 |  |
| **BMI, kg/m^2^** |  |  |  |  |  | 0.157 |
| <25 | ref | 0.58(0.34,0.99) | 0.89(0.51,1.57) | 0.58(0.34,1.01) | 0.164 |  |
| 25-30 | ref | 1.24(0.86,1.78) | 0.84(0.55,1.26) | 0.84(0.55,1.27) | 0.15 |  |
| >30 | ref | 0.80(0.55,1.16) | 0.96(0.62,1.49) | 0.76(0.50,1.15) | 0.344 |  |
| **Smoking status** |  |  |  |  |  | 0.285 |
| never | ref | 1.00(0.67,1.48) | 0.93(0.66,1.32) | 0.64(0.43,0.95) | **0.013** |  |
| former | ref | 0.82(0.46,1.46) | 0.92(0.57,1.46) | 0.98(0.64,1.49) | 0.825 |  |
| now | ref | 0.64(0.36,1.13) | 0.94(0.49,1.79) | 0.69(0.43,1.12) | 0.35 |  |
| **Alcohol use** |  |  |  |  |  | 0.277 |
| never | ref | 0.90(0.46,1.77) | 1.14(0.59,2.20) | 1.04(0.55,1.98) | 0.718 |  |
| former | ref | 0.94(0.63,1.40) | 1.16(0.69,1.92) | 0.52(0.32,0.86) | 0.091 |  |
| now | ref | 0.79(0.57,1.09) | 0.78(0.55,1.12) | 0.72(0.53,0.98) | 0.057 |  |
| **Hypertension** |  |  |  |  |  | 0.711 |
| no | ref | 0.83(0.59,1.16) | 0.82(0.59,1.14) | 0.75(0.54,1.05) | 0.116 |  |
| yes | ref | 0.83(0.56,1.21) | 0.97(0.65,1.45) | 0.68(0.50,0.92) | **0.04** |  |
| **Diabetes** |  |  |  |  |  | **0.032** |
| no | ref | 0.77(0.57,1.04) | 0.84(0.60,1.18) | 0.61(0.47,0.80) | **0.003** |  |
| yes | ref | 1.14(0.76,1.72) | 1.13(0.70,1.83) | 1.45(0.84,2.52) | 0.195 |  |
